# Breeding Rice to Increase Anthocyanin Yield Per Area through Small, Black Grain Size and Three Grains per Spikelet

**DOI:** 10.3390/plants13192713

**Published:** 2024-09-27

**Authors:** Thanarote Sricha, Tidarat Monkham, Jirawat Sanitchon, Myo San Aung Nan, Teerawat Suwannual, Sompong Chankaew

**Affiliations:** 1 Department of Agronomy, Faculty of Agriculture, Khon Kaen University, Khon Kaen 40002, Thailand; thanarote.s@kkumail.com (T.S.); tidamo@kku.ac.th (T.M.); jirawat@kku.ac.th (J.S.); myo081159@gmail.com (M.S.A.N.); 2 Khon Kaen Rice Research Center, Muang District, Khon Kaen 40000, Thailand; teerawat.s@rice.mail.go.th

**Keywords:** anthocyanin content, anthocyanin yield, grain surface area, pre-breeding

## Abstract

Rice varieties with high anthocyanin content are often recognized for their vibrant colors and health benefits. The demand for rice with high anthocyanin is increasing domestically and internationally due to consumers becoming more health-conscious. However, the current increase in yield might not raise the anthocyanin content due to its location in the grain pericarp and seed coat, which are relative to the grain surface area. This study aims to develop rice lines to increase anthocyanin yield per production area by improving rice varieties with small, black, and three grains per spikelet. Accordingly, six rice recombinant inbred lines (RILs) were bred by crossing Niaw Dam Chaw Mai Pai 49 (NDCMP49) with Khao Nok (LLR059). The grain color, size, and number of grains per spikelet were selected from the F_1_ to the F_4_ population through the pedigree selection method. Six RILs and their parents were assigned in a randomized complete block design (RCBD) with three replications under field conditions during the rainy season of 2021 and 2022 in four locations. The results showed statistically significant differences in environmental conditions, affecting productivity and the yield components of rice lines. Consequently, the rice lines adapted to a specific environment, and there were significant differences in genotype. This study identified three RILs with higher yield performance (13-1 (3842 kg/ha), 374-1 (3699 kg/ha), and 903-3 (3550 kg/ha)) compared with the parent NDCMP49 (1996 kg/ha). However, the grain yields were unstable in the three top-yielding RILs due to varying environmental conditions, indicating that selective breeding requires a specific, narrow environment. Based on grain yield and grain size, the RILs performed better in the grain surface area than in the parent NDCMP49. Moreover, only two RILs (374-1 and 903-3) produced the highest anthocyanin content and yield, although this was lower than in the parent NDCMP49. However, the 374-1 and 903-3 RILs produced more grains, black grains, and three grains per spikelet with high yield and moderate anthocyanin content. They can, therefore, be backcrossed to the parent NDCMP49 to increase the accumulated anthocyanin content with a stable, high yield. This work provides a resource of small grains, black grains, and three grains per spikelet in the rice breeding line for breeding programs in the future.

## 1. Introduction

Climate change and population expansion are increasing the demand for animal feed and biofuels. As a result, rice production significantly impacts food security and the socioeconomic position of more than half of the world’s population [1]. Each rice genotype is affected by constraints such as biotic and abiotic stress, causing yield uncertainty. Many countries seek to increase rice productivity in rice field management or develop new, high-yielding rice varieties. As a result, new rice types providing high yields that match the requirements must be developed through breeding in conjunction with technological advancements in genetics, genomics, and phenomics [2].

Purple or black rice containing anthocyanin is recognized as a source of natural antioxidant compounds among health-conscious rice consumers as their staple food [3]. Anthocyanin in rice has been reported to benefit human health for its antioxidant properties, protection of endothelial cells, prevention of cardiovascular disease, anti-cancer effect, diabetes control, and visual acuity recovery [4,5]. Anthocyanins are secondary compounds in plant defense mechanisms under stressful conditions [3,6]. In general, anthocyanin accumulation is controlled in the presence of biological and abiotic stress. In addition, the mechanism of anthocyanin accumulation is controlled by many genes [7]. Anthocyanin content in rice is associated with the grain surface area due to its accumulation in the pericarp and seed coat layers of black rice [8,9,10]. Also, about 97% of total anthocyanin content (TAC) is present in the pericarp, seed coat, aleurone layer, and bran of black rice caryopsis, and around 3% is present in the embryo [8,9,10]. An increase in the grain surface area might enhance the anthocyanin content in rice. However, increasing the grain surface area usually depends on the number of grains per panicle and grain size.

Grain size is a quantitative trait controlled by multiple genes [11], whose grain size relative to the grain surface area is an essential factor affecting the quality of the grain and market value [12,13]. Increasing the grains per panicle and grain surface area is a crucial target for rice breeding. In addition, grain size can also be used to classify rice types that reflect the different rice consumption patterns in each area [11,12,13]. Rice is categorized into three extensively farmed ecological varieties: long-grained indica rice, produced in tropical and subtropical Asia; medium-grained japonica rice, grown in the Philippines and hilly areas of Madagascar and Indonesia; and short/medium-grained japonica rice, grown in temperate regions like Japan and northern China [14]. Currently, rice farmers favor rice with long [15], slender grains, and many grains per panicle. However, there is also a lack of asymmetry between sink and source [16]. As a result, it is impossible to reduce the number of filled grains per panicle to increase yield and the grain surface area by using larger grains. Grain size is an important physical indicator of quality and is frequently related to yield [17] and grain surface area. Small grains possess a larger surface area than large grains when the same production is compared [18]. Again, grain size and surface area influence the anthocyanin content due to its accumulation in the pericarp or seed coat and aleurone layers [8,9].

The appearance of the inflorescence determines reproduction and productivity [19], and therefore, the number of grains per inflorescence/spikelet significantly influences the grain yield potential of the crops [20,21,22,23]. A study on wild rice florets was found to be deterministic. It produced a single fertile floret from a mutant of three grains per inflorescence when “two florets” and “three florets” were genetically selected [20,21,22]. This indicates that the incomplete development of florets may share a common genetic basis in plant inflorescence. Deciphering the molecular regulators controlling flower and flower fertility is crucial to the grain number and yield. Aung Nan [23] studied the inheritance of three grains per spikelet from Thai native rice germplasm, Niaw Dam Chaw Mai Pai 49 (NDCMP49), controlled through monogenic inheritance with incomplete dominance. This indicates that transferring such features to different rice cultivars is thus possible. Therefore, three grains per spikelet also increases the number of grains per panicle [23]. The three grains per spikelet and small grain size are traits for early visual selection before pollination in an efficient breeding program via the conventional breeding method. Furthermore, important agronomic traits such as plant architecture should be selected by considering the sink-source balance influencing the complete grain filling to increase the number of grains per panicle.

Therefore, this study aims to generate rice RILs that increase the anthocyanin yield per production area by improving the small-grain LLR059 variety with the black grains and three grains per spikelet of the NDCMP49 variety. This study provides valuable rice RILs with desirable traits for future rice breeding.

## 2. Results

### 2.1. Evaluation of Traits in Rice RILs and Their Parents under Multi-Location Conditions

A combined analysis of variance was used to evaluate 1000 grain weight (1000 GW), grain yield, grain surface area per 1000 grains (GSA/1000 grains), grain surface area per hectare (GSA/ha), anthocyanin content, anthocyanin yield, and harvest index (HI). Analysis among RILs revealed high significance (*p* < 0.01) for all traits. Grain yield, 1000 GW, anthocyanin content, and HI presented highly significant differences among the various environmental conditions. However, the G×E interactions were highly significant for grain yield, 1000 GW, GSA, anthocyanin content, and HI (Table 1). The results demonstrate that the responses of the improved rice RILs to the traits mentioned above vary depending on the environment (Table 1). Grain yields and the anthocyanin yield of eight genotypes in four environments are summarized in Table 2. The RILs produced a higher yield than the NDCMP49 variety, although the anthocyanin yield was still lower than that of the NDCMP49 variety (Table 2).

The main objective of this study is to compare the performance of RILs with their parents. The combined mean values for grain yield, 1000 GW, GSA/1000 grains, GSA/ha, anthocyanin content, anthocyanin yield, and HI across four field environments of the RILs and their parents are presented in Table 3. As can be observed, the RILs and their parents show significant differences in all evaluated traits (Table 3). The 1000 GW of RILs mean values range from 13.16–16.45 g, smaller than that of NDCMP49 (28.56 g), while the grain yield values of RILs range from 2905–4214 kg/ha, higher than that of NDCMP49 (1996 kg/ha). The results indicate that the small grain trait from the LLR059 parental line can enhance the grain yield in RILs. All RILs have mean values of GSA/1000 grains, ranging from 2661.60–3275.10 cm^2^/1000 grains, less than those of NDCMP49 (5559.90 cm^2^/1000 grains). Interestingly, when considering the GSA/ha, the small grain size RILs significantly differed from NDCMP49 (Table 3). The results demonstrate that the selected RILs with small grain sizes and high yield contribute to the high GSA/area because the number of grains is higher when the yield is comparable.

The anthocyanin content in the mean values of RILs ranged from 1.45–17.69 mg/100 g grain, lower than those of NDCMP49 (68.78 mg/100 g grain) (Table 3). The two RILs with high anthocyanin content were lines 374-1 (17.69 mg/100 g grain) and 903-3 (11.78 mg/100 g grain), although this was still approximately 4–5 times less than the parent variety NDCMP49. The results indicate that the selected RILs might not accumulate all combinations of anthocyanin genes during the breeding and selection. However, RILs 374-1 and 903-3 exhibited an anthocyanin yield comparable to approximately 50% of the parent variety (NDCMP49). The results indicate that the anthocyanin yield can be enhanced by combining anthocyanin content, GSA/ha, and yield (Table 3). In general, the RILs selected from this study met the criteria of small grains, black grains, and three grains per spikelet characteristics for improving the anthocyanin yield of rice.

### 2.2. General Genotypic Adaptation

#### Genotype Plus Genotype by Environment Interaction Biplot Analysis (GGE Biplot)

**Figure 1:**
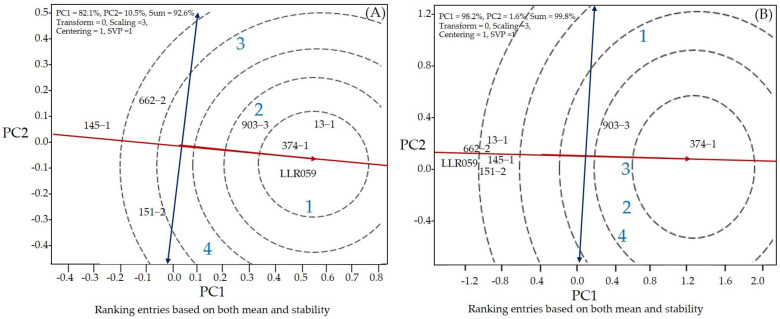
The GGE biplot of six rice hybrid combinations in four locations for grain yield (**A**) and anthocyanin content (**B**). (1) Khon Kaen Rice Research Center in 2021; (2) Khon Kaen Rice Research Center in 2022; (3) Khon Kaen University Field, Thailand; and (4) Ban Haet District, Khon Kaen Province, Thailand.

GGE biplots were used to interpret the general genotypic adaptability of Niaw Dam Chaw Mai Pai 49, LLR059 (Khao Nok), and RILs. The GGE biplot results show a total variation in the grain yield of 92.6%, comprising PC1 (82.1%) and PC2 (10.5%) (Figure 1A). The PC1 score indicates the grain yield of the lines: PC1 > 0 indicates the high grain yield lines, whereas PC1 < 0 indicates the low grain yield lines. The PC2 score derived from the multi-location tests indicates the line’s stability. If the PC2 score approaches zero, the lines are stable. Based on the GGE biplot analysis, the rice line scores for PC1 > 0 and low PC2 were RILs 13-1, 374-1, and 903-3, indicating that the lines exhibited high yield and high stability.

The total variation in the anthocyanin content was 99.8%, comprising PC1 and PC2 values of 98.2% and 1.6%, respectively (Figure 1B). The lines demonstrating high anthocyanin content (PC1 > 0) were RILs 374-1 and 903-3, while 13-1, 145-1, 151-2, and 662-2 exhibited average stability (low PC2 score). RILs 374-1 and 903-3 had the highest mean grain yield and anthocyanin content (Table 3). Identification of the ideal genotype for grain yields and anthocyanin content revealed that RILs 374-1 and 903-3 were positioned the closest to the ideal grain yields and anthocyanin content lines (Figure 1). RILs 374-1 and 903-3 were indicated to be the ideal small-grain rice line for high stability, high grain yields, and anthocyanin content (Table 3 and Figure 1).

**Figure 2:**
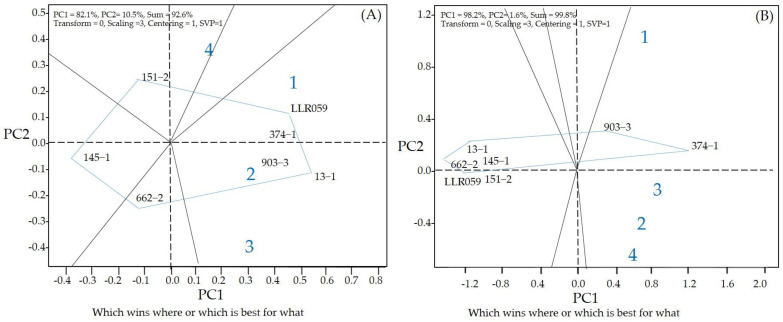
Polygon views of the GGE biplot of the “which-won-where/what” pattern of genotypes and locations. Grain yield (**A**) anthocyanin content (**B**). (1) Khon Kaen Rice Research Center in 2021; (2) Khon Kaen Rice Research Center in 2022; (3) Khon Kaen University Field, Thailand; and (4) Ban Haet District, Khon Kaen Province, Thailand.

The grain yield analysis for the rice lines studied at multiplications was facilitated using a “which-won-where” pattern, showing the interaction of genotype to the datasets of different environments at the multi-location yield trials. The polygon view of this biplot indicates the test locations in five sectors, with the lines at the corner of each section having the highest yield. The polygon view shows that the RIL 13-1 had the highest grain yield (1) in the Khon Kaen Rice Research Center in 2021, (2) in the Khon Kaen Rice Research Center in 2022, and (3) in the Khon Kaen University Field, Thailand. 151-2 had the highest grain yields in (4) Ban Haet District, Khon Kaen Province, Thailand (Figure 2A).

Considering each location, the RILs 374-1 and 903-3 had the highest anthocyanin content at (1) Khon Kaen Rice Research Center in 2021; (2) Khon Kaen Rice Research Center in 2022; (3) Khon Kaen University Field, Thailand; and (4) Ban Haet District, Khon Kaen Province, Thailand (Figure 2B).

Therefore, from the GGE biplot study on the yield characteristics and amount of anthocyanin in the breeding rice, lines 374-1 and 903-3 showed high yield potential and anthocyanin content. The traits of small grains, black grains, and three grains per spikelet allowed the anthocyanin content to be increased. However, when comparing the performance of the improved line with that of NDCMP49, the latter was found to have the lowest yield and the highest anthocyanin content (Table 3). Consequently, when examining stability, the NDCMP49 rice variety could not be classified.

## 3. Discussion

Choosing appropriate parents to create successful hybrid lines creates a significant obstacle for rice breeders. The goal is to achieve elevated yields and enhanced rice grain quality. The capacity to blend parental strains with unique traits is a valuable attribute that effectively enhances rice production. Rice yield, a multifaceted characteristic, hinges on three primary factors: grain weight, quantity of inflorescence per plant, and number of grains per inflorescence [12,24]. Rice inflorescence is crucial in influencing rice grain yield [25]. This study aims to improve rice varieties with the characteristics of black grains, small grains, and three grains per inflorescence. The experimental results indicate successfully transmitting these desired characteristics to the offspring, as Table 2 and Table 3 outlined. This study shows that visual selection of grain size and three grains per spikelet before mating shortens the time required to develop rice varieties. Furthermore, since the parents’ genetics control these characteristics to create the population, it is inherited from the parents into the F_1_ population. In contrast, the three desired characteristics are displayed in the F_2_ population with the highest genetic distribution, aligning with the established principles of rice breeding. This study explicitly investigates the trait transmission of three seeds per spikelet, controlled by a single dominant gene displaying incomplete suppression [23]. Nevertheless, when attempting to combine the attributes of purple rice, characterized by the presence of anthocyanin, the transmission of this particular trait could not be enhanced. Two RILs (374-1 and 903-3) had the highest anthocyanin content but were still lower than in the parent variety NDCMP49 (Table 3). This restriction resulted from the parents, NDCMP49 and LLR059 (Khao Nok), having distinct genetic makeups for black and white-brown rice grains, respectively. Furthermore, the early-generation seed color selection did not undergo precise analysis of the anthocyanin content. That might cause a loss of anthocyanin gene combination in the RILs due to the mechanism of anthocyanin accumulation being controlled by many genes (quantitative characteristics) [7]. However, the RILs with small grain sizes and three grains per spikelet were successfully selected. Therefore, the RILs should be backcrossed to the parent NDCMP49 to increase the accumulated anthocyanin content with a stable, high yield.

**Figure 3:**
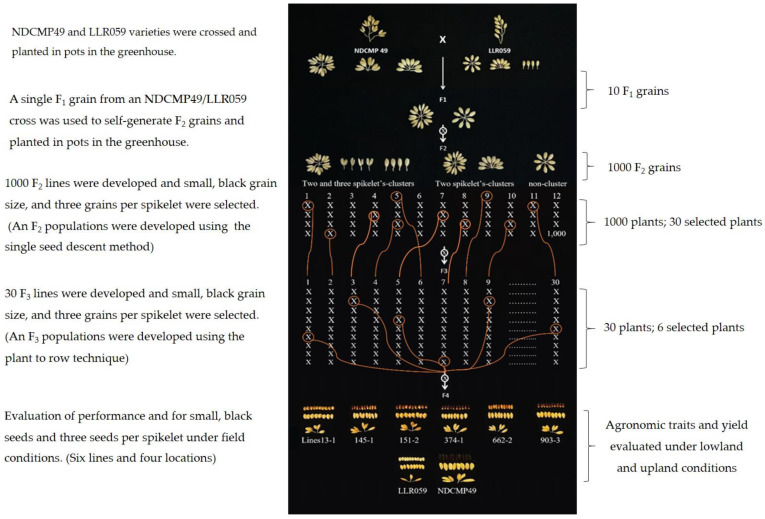
Population development of recombinant inbred lines (RILs) via the pedigree method.

Rice brown grain colors are generally divided into red, green, black, and white varieties, and the composition of anthocyanin pigments was different in determining their color [26]. Different primary anthocyanin pigments were identified in colored rice cultivars, the cyanidin-3-glucoside (C3G) and peonidin-3-glucoside (P3G) [27,28], petunidin-3-glucoside (Pt3G) [29] were identified as primary anthocyanin pigments of black rice. In contrast, the primary anthocyanin pigments are unclear in red rice. Abdel-Aal et al. [27] reported that C3G was the main anthocyanin in red rice. In contrast, Kim et al. [30] and Finocchiaro et al. [31] concluded that red rice did not contain anthocyanin pigments. Moreover, Abdel-Aal et al. [27] found that total anthocyanin content (TAC) differed significantly among black rice cultivars. The anthocyanin content of black and red-brown rice was recently published by Laokuldilok et al. [32] and Chen et al. [26], and it seems that the TAC of colored rice increases as the color deepens. This study classified the RILs into brown, light brown, slight, speckled brown, and variable purple (Figure 3 and Table S1). The results are consistent with the reports of Maeda et al. [33], who reported that the RILs divided from the cross between the parents with black- and white-brown rice grain colors are classified as black, brown, partial brown, light brown, and white-brown rice grain colors. Lap et al. [34] reported that the anthocyanin content of selected RILs with different brown rice colors, such as light brown, dark brown, reddish-purple, and purple, are different.

Rice is the staple food for over half the world’s population [35]. To address the escalating global demand for food, improving rice varieties to achieve higher yields or enhancing quality by leveraging genetic diversity, particularly in local rice varieties harboring traits critical to developing novel rice varieties, is necessary. While traditional rice production concentrates on inflorescence morphology, rice breeders have not focused on specific factors like the number of branches, inflorescence length, and flower density and consider the number of florets in each flower [20]. The rice breeding approach focuses on achieving smaller grains and a target of three grains per inflorescence, leading to a notable enhancement in rice yield (Table 3). This increase is attributed to more grains per spikelet, with three grains contributing significantly to the overall grain count. Furthermore, the yield component, specifically the number of shoots per clump and inflorescence per cluster, is crucial in augmenting the yield per unit area, as illustrated in Table S2. This is consistent with the work of Saroj et al. [36], who studied the components of variability and heritability in Indian mustard [*Brassica juncea* (L.) Czern. and Coss.] and found that in biological production, the characteristics of total grain yield, plant height, number of panicles, date of flowering, plant height at maturity, number of shoots, and grain size play a crucial role in grain yield per plant.

Reducing the seed size has proven beneficial in achieving yields that are either higher or comparable to the original yields, as observed in the case of the LLR059 (Khao Nok) variety. This variety demonstrates a superior yield compared to NDCMP49 due to its smaller grains. The enhancement in rice varieties with smaller grains contributes to sustainable food production, aligning with the principles of creating a balance between food generation and storage. This observation is in line with the research conducted by Venkateswarlu [37] and Shi et al. [38] regarding the interplay between food production and rice accumulation. These studies indicate that rice grain production assimilates supply (source capacity) while grain capacity assimilates demand (sink strength). Moreover, rice varieties can be divided into types of relationships with food-generating constraints. Types of food storage restrictions and interaction between food production and food storage sites [39], since sufficient capacity for food storage and sound production and storage relationships are essential for high rice yield [40,41]. Additionally, the timing of rice grain replenishment holds significant importance in determining rice grain yield [42]. Ensuring sufficient food production during this critical stage, derived from non-structural carbohydrate reserves, is crucial. Non-structural carbohydrates (NSC) play a pivotal role in accumulating an ample food supply to achieve high yields while ensuring good-quality rice [43,44].

**Figure 4:**
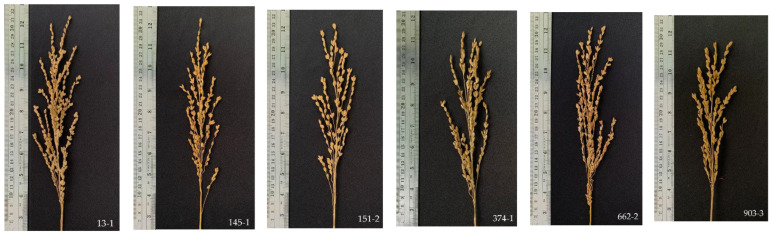
Rice panicle and grain filling characteristics of six improved rice varieties (lines 13-1, 145-1, 151-2, 374-1, 662-2, and 903-3).

Most contemporary rice traits have been selectively bred to exhibit favorable food storage attributes, exemplified by features like more spikelets per panicle [45,46]. Concurrently, there is a focus on enhancing food production characteristics, including higher yield, increased dry weight [45], and an elevated leaf area index [47]. Furthermore, the harvest index serves as an indicator of resource allocation efficiency within the food production domain. In this investigation, rice variety yield potential enhancement is derived from initial-generation observations for variety selection (Figure 4) and harvest index (HI) values. All six selected varieties displayed commendable characteristics, with particular emphasis on the notable efficiency of line 13-1 in achieving a balanced food production source. Conversely, line 662-2 exhibited consistently low HI values across various environments (Table 3 and Table S3–S6), indicating inappropriate management of the rice plant’s food resources. This emphasizes the need to improve food storage and source potential concurrently, referred to as the source–sink relationship. To attain elevated seed replenishment and amplify yield potential, enhancements in food storage, including the number of grains per inflorescence, grain weight, and complete grain filling, must be coupled with improvements in the plant’s ability to serve as a food source through effective carbohydrate supply [48].

In this research, the LLR059 (Khao Nok) and NDCMP49 varieties were used as parents for use in developing lines of rice varieties to augment yield and elevate to augment yield and elevate anthocyanin content. This was achieved by increasing the number of grains per inflorescence to three, incorporating smaller black and brown rice grains, and promoting thorough grain filling. The study findings reveal that cultivating smaller rice grains contributes to increased grains per inflorescence, enhancing productivity or maintaining consistent production levels. Additionally, smaller grain sizes result in an expanded surface area for the grains. The smaller size allows for more grains per inflorescence, leading to an augmented surface area than larger grains of equivalent weight (Table 3). When assessing grain size, it is evident that the surface area of rice grains increases compared to larger grains with similar weights. Consequently, the selection of smaller grains contributes to stable or increased yields and enhances the overall surface area of the grains. A higher grain surface area increased anthocyanin content per unit area. This relationship is evidenced in Table 3, where the calculated amount of anthocyanin per area demonstrates an increase associated with a larger grain surface area.

In this experiment, rice varieties grown in different environments (Table 4) caused grain yield and anthocyanin yield to be different in each area due to the accumulation of anthocyanin in each rice variety is influenced by various factors such as light, temperature, soil pH, management practices (including light shade), UV, and, notably, genetics. Among these factors, genetics holds particular significance since anthocyanin accumulation is intricate and regulated by the interactions of multiple gene groups, which could alter the expression of anthocyanin synthesis genes and accumulations [7,49,50]. The presence of different alleles in numerous genes associated with qualitative traits results in the manifestation of diverse phenotypes. For instance, *OsC1* has been identified as a potential activator of the DFR and ANS involved in anthocyanin production. In other plant organs, anthocyanins mainly serve as pigments in stems [51]. Additionally, the *OsBBX14* and *OsHY5* transcription factors have been found to possess decoding-stimulation activity, thereby influencing anthocyanin synthesis by controlling the *OsC1* and *OsB2* genes [52]. Consequently, these genes may coordinate or operate independently in anthocyanin synthesis, leading to variations in anthocyanin accumulation across different regions.

This study reveals variations in the environmental conditions of each test plot, encompassing differences in soil properties, geographic characteristics, and rainfall amounts (Figure S1 and Table 4). These differences have notable impacts on yield, production components, and the quantity of anthocyanin substances, with each species responding differently to its specific environment. Beyond geographic distinctions, rainfall distribution, as illustrated in Figure S1, is a crucial factor influencing the cultivation process and crop production. A case in point is the rice fields in Ban Nong Saeng, Nong Saeng Subdistrict, Ban Haed District, Khon Kaen Province, where even rainfall distribution contributes to the successful yield of improved rice strains. Furthermore, field testing serves as an initial assessment to explore the stability of the enhanced strains tailored to specific regions. From the results in Table 2, three genotypes, 13-1, 374-1, and 903-3, gave high yields, but yields are still unstable. This is due to varying environmental conditions regarding chemical composition, soil physics, climate, soil texture, meters above sea level, temperature, and rainfall amount, as shown in Figure S1 and Table 4, indicating that selective breeding requires a specific, narrow environment. Notably, the 151-2 variety demonstrates adaptability and high yield in rice cultivation areas, emphasizing the specificity of the six improved strains to different environments. This specificity results from the crossbreeding between the mother variety NDCMP49, adapting to field rice conditions, and the LLR059 (Khao Nok) variety suitable for lowland rice cultivation. Leveraging the distinct advantages of parent breeds, genotypes 374-1 and 903-3 exhibit versatility in cultivation in lowland (Tables S3 and S4) and upland conditions (Tables S5 and S6). This dual adaptability offers the potential to enhance production and anthocyanin levels in rice, making these genotypes advantageous candidates for future varieties. Strategies for further increasing anthocyanin content in the future include the development of the 374-1 and 903-3 RILs, producing a more significant number of small grains, black grains, and three grains per spikelet with high yield and moderate anthocyanin content and can, therefore, be backcrossed to the parent NDCMP49 to increase the accumulated anthocyanin content with a stable high yield. This work provides a resource of small grains, black grains, and three grains per spikelet in the rice breeding line for breeding programs in the future.

## 4. Materials and Methods

### 4.1. Plant Materials

This study aims to improve rice varieties to achieve high anthocyanin content by increasing the number of grains per plant and three-grain inflorescence. Two parents were used in this study: NDCNP49, a colored rice variety local to Thailand’s southern region, collected by the Pattani Rice Research Center since 1996 [23], and LLR059, a small grain variety obtained from the Rice Project, Khon Kaen University, Khon Kaen, Thailand.

To breed a hybrid first-generation (F_1_) population with 10 seeds, the cultivated F_1_ population is used to self-fertilize until F_2_ generation (F_2_) populations are obtained [23]. The F_2_ seeds (1000 seeds) were utilized to generate the population using the single-seed descent technique to select F_2_ populations with desirable features. The appearance of inflorescence at 1, 2, and 3 grains per inflorescence (two and three spikelet clusters) was achieved by considering the formation of inflorescence, namely 1 grain (non-cluster), 1 and 2 grains (two spikelet clusters) and 1, 2, and 3 grains per inflorescence (two and three spikelet clusters), grain size, and grain coat color via visual selection and selecting 30 lines out of 1000 (Figure 3).

Thirty selected F_3_ lines were planted using the plant-to-row technique (10 plants/row/line). The plants in each row were chosen for two and three spikelet clusters, small grains, and a black grain color when obtaining relatively high genetic stability of six lines. In the F_4_ population, the details of selected lines as 13-1, 145-1, 151-2, 374-1, 662-2, and 903-3 were shown in Table S1. The results demonstrate the effective transmission of the traits for three grains per inflorescence, small grain size, and black grain color through breeding. The three grains per inflorescence and small grain size were successful introgressions to RILs, while the color of brown rice grains of RILs was not similar to NDCMP-49. The color of brown rice grains of each RIL is light brown (13-1), speckled brown (145-1 and 151-2), brown (662-2), and variable purple (374-1 and 903-3) (Figure 3 and Table S1). To assess these outcomes, the yield and anthocyanin content of the selected population were compared to those of the black sticky cultivar NDCMP49 and LLR059 variety (Figure 3).

### 4.2. Experimental Design/Field Management/Agricultural Practice

Six recombinant inbred lines (13-1, 145-1, 151-2, 374-1, 662-2, and 903-3) of F_4_ generation from the Niaw Dam Chaw Mai Pai 49 × LLR059 (Khao Nok) cross and their parents were used in this study. The experiments were conducted under lowland conditions at (1) Khon Kaen Rice Research Center in 2021 (16°25′46 N, 102°49′16 E); (2) Khon Kaen Rice Research Center in 2022 (16°25′46 N, 102°49′16 E); (3) Khon Kaen University Field, Thailand in 2022 (16°28′19 N, 102°48′34 E); and under upland conditions at (4) Ban Nong Saeng, Ban Haet District, Khon Kaen Province, Thailand in 2022 (16°10′04 N, 102°49′57 E). All entries were assigned in a randomized complete block design (RCBD) with three replications each year and at every location during the rainy season. The information of four experimental fields is exhibited in Table 4.

#### Field Validation

(1) Lowland conditions

Six selected RILs were evaluated for yield, yield components, stability, and anthocyanin content, with two check varieties including donor parent Niaw Dam Chaw Mai Pai 49 and recurrent parent LLR059 (Khao Nok). For the field evaluation, 30-day seedlings were selected for transplantation; one seedling was used on one plant per hole. The plot size was 3.0 m × 4.0 m (256 plants per plot) with row and hill spacing of 25 and 25 cm, respectively. Fertilizer was applied at a rate of 25 kg/ha:31 kg/ha:0 kg/ha (N:P_2_O_5_:K_2_O) 30 days after planting, and weeding performed at the same time, with a top-dressing of 72 kg N/ha applied 60 days after planting. Plants were protected through weeding and chemical treatments at recommended rates from the onset of outbreaks until harvest to prevent the spread of disease, insects, snails, and crabs. Field water was maintained during the tillering stage at around 5–10 cm until ten days before harvesting.

(2) Upland conditions

A total of 6 RILs and 2 check lines were evaluated: a donor parent and a recurrent parent. The plots were directly seeded using 3–5 seeds per hill, with subsequent thinning to 1 seedling per hill at 10–15 days after the seedlings emerged. The plot size was 3.0 m × 4.0 m (160 plants per plot) with row and hill spacing of 40 and 25 cm, respectively. Fertilizer was applied at a rate of 23.4 kg/ha:23.4 kg/ha:23.4 kg/ha (N:P_2_O_5_:K_2_O) at 30 days after seedling emergence, and weeding was performed at the same time, with a top-dressing of 72 kg N/ha at 60 days after seedling emergence. Plants were protected by hand weeding and the use of common pesticides.

These findings underscore the pivotal role of environmental conditions and plant growth in optimizing rice cultivation efficiency (Table 4).

### 4.3. Data Collection

Data on rice yield, yield components, and agronomic characteristics, such as grain yields, were recorded at 1 m^2^ from the inner rows of each plot and then weighed. The 1000-grain weight (1000 GW) was measured. Grain width, length, and thickness were measured using vernier calipers. The geometric means of the three basic seed dimensions were then determined using the connection Equation (1) proposed by Shkelqim et al. [53]. The grain surface area (GSA) was then calculated by analogy with a sphere having a geometric mean diameter using Equation (2) proposed by Jouki and Khazaei [54].

D_g_ = [L × W × T]^1/3^ (1)

where D_g_ is the geometric mean diameter (mm), L is grain length (mm), W represents grain width (mm), and T is the grain thickness (mm).

GSA = π D_g_^2^ (2)

where GSA (mm^2^) represents the grain surface area, and D_g_ stands for geometric mean diameter (mm), respectively.

The total grain and plant dry weights were randomly measured on four plants/sub-plots. The randomly selected plants were cut to determine aboveground dry weight at the harvesting stage. Sun drying was applied to reduce the humidity of the rice plants for about 3–5 days. The aboveground total dry weight (TDW) was then measured using a balance. Following threshing, the grain yield was recorded. The harvest index (HI) was calculated using the formula (HI) = total grain weight/total plant dry weight. The anthocyanin content was measured using four plants within the inner rows of each subplot. The anthocyanin content of the parents and six RILs was measured in the laboratory by a spectrophotometric method adapted from Lee et al. [55]. After harvesting, the seeds were cleaned and dried, and samples of rice grains from each variety were manually hulled and ground to obtain a fine powder using a cyclone mixer mill (HMF-590; Hanil, Seoul, Korea) with a mortar and pestle to get rice flour (starch). About 0.2 g of freeze-dried sample was added into a 50 mL tube, and 25 mL of ethanol-0.1% TFA (trifluoroacetic acid) solution was added to the sample. The reaction was allowed to proceed in the dark at 4 °C for 24 h, and then the extract was centrifuged at 4000 rpm for 10 min. Pour the extract into an Erlenmeyer flask. Adjust the amount of extract to 25 mL. Then, the sample was extracted with a 0.45-micron nylon membrane filter once and used to measure the anthocyanin content by spectrophotometry at 520 nm. The following formula calculated the total anthocyanin content (3) [55].

MAP = [(A × MW × DF)/(ε × l)] × 1000(3)

when

MAP = Monomeric anthocyanin pigment
A = The absorbance at a wavelength of 520 nm
MW = Molecular weight of cyanidin 3-glucoside (449.2 g/mol)
DF = The dilution factor is l (final volume/initial volume)
l = cell path length in cm (1 cm)
ε = 26,900 molar extinction coefficient, in L × mol^−1^ × cm^−1^
1000 = factor for conversion from g to mg

### 4.4. Data Analysis

Data on the agricultural characteristics, yields, yield components, and anthocyanin content of lines and varieties were analyzed via the STATISTIC 10 program (Copyright^©^ 1985–2013, Analytical Software 2105 Miller Landing Rd. Tallahassee, FL, USA), and means compared by the least significant difference (LSD) and GGE Biplot [56].

## 5. Conclusions

To increase anthocyanin yield per production area, the rice variety was improved by using small seeds, black seeds, and three seeds per spikelet characteristics and crossing Niaw Dam Chaw Mai Pai 49 with LLR059 (Khao Nok) via the pedigree method. Three high-yielding genotypes were identified: 13-1 (3842 kg/ha), 374-1 (3699 kg/ha), and 903-3 (3550 kg/ha). However, these three top-yielding genotypes produced unstable grain yields under various environments since they were each narrowly adapted to a specific environment. Moreover, only two genotypes produced the highest anthocyanin content, RILs 374-1 and 903-3. These inbred lines provided resources of small seeds, black seeds, and three seeds per spikelet for rice breeding programs in the future.

## Figures and Tables

**Table 1:** Summary of ANOVA results showing mean square values for grain yield, 1000 GW, GSA/1000 grains, GSA/ha, anthocyanin content, anthocyanin yield, and HI variable for four environments/locations in combination.

Source of Variation	df	Grain Yield	1000 GW	GSA/1000 Grains	GSA/ha	Anthocyanin Content	Anthocyanin Yield	HI
Envi. (E)	3	3,385,456 *	7.94 **	25,861 ns	9.48 × 10^12^ **	609.26 **	7.45 × 10^9^ **	0.0247 **
Rep./Envi.	8	743,747	0.29	26,761	1.93 × 10^12^	0.12	1.59 ×10^8^	0.0007
Genotype (G)	7	4,331,793 **	316.63 **	1.24 × 10^7^ **	1.47 × 10^13^ **	6432.29 **	2.78 × 10^10^ **	0.0168 **
G×E	21	381,833 **	3.13 **	24,092 *	1.96 × 10^12^ **	263.60 **	2.18 × 10^9^ **	0.0017 **
error	56	86,085	0.21	11.23	3.76 × 10^11^	0.30	8.66 × 10^7^	0.0004
C (%)		9.07	2.77	3.34	10.08	4.04	26.41	5.99

** = significantly different at *p* < 0.01, * = significantly different at *p* < 0.05, ns = non-significant at *p* > 0.05. CV = coefficient of variation, 1000 GW = 1000 grain weight, GSA/1000 grains = grain surface area per 1000 grains, GSA/ha = grain surface area per ha, and HI = harvest index.

**Table 2:** Grain yields and anthocyanin yields of eight rice genotypes in each environment and under four environmental fields.

Lines/Variety	Grain Yields (kg/ha) *				Anthocyanin Yield (mg/ha)			
	KKRRC 2021	KKRRC 2022	KKUA4 2022	BH 2022	KKRRC 2021	KKRRC 2022	KKUA4 2022	BH 2022
13-1	4214	3348	4536	3270	8267	4335	7770	5267
145-1	2546	2815	3327	2931	6686	6335	13,936	9614
151-2	3347	2634	3208	3410	5105	8.279	12,624	9033
374-1	4012	3271	4186	3325	54,795	47,104	121,253	45,393
662-2	3104	2720	3883	2672	3655	3848	8189	2958
903-3	3906	3020	4167	3108	43,843	26,314	72,582	30,166
LLR059	4341	3390	3817	3056	1493	2897	3899	2687
NDCMP49	1663	1522	2337	2461	89,521	99,829	252,567	117,268
Envi. mean	3391	2840	3683	3029	26,671	24,868	61,603	27,798

* KKRRC = Khon Kaen Rice Research Center, KKUA4 = Khon Kaen University A4 field, BH = Ban Haet District, Khon Kaen Province.

**Table 3:** Grain yield, 1000 GW, GSA/1000 grains, GSA/ha, anthocyanin content, anthocyanin yield, and HI of RILs and their parents (NDCMP49 and LLR059), tested in four experimental fields of 2021–2022.

Lines/ Variety	Grain Yield (kg/ha)	1000 GW (g)	GSA (cm^2^/1000 Grains)	GSA (cm^2^/ha)	Anthocyanin Content (mg/100 g Grains)	Anthocyanin Content/Area (mg/ha)	HI
13-1	3842 a	16.45 b	3121.40 c	7.21 × 10^8^ a	1.64 e	6410 d	0.36 a
145-1	2905 d	13.16 e	2661.60 e	5.83 × 10^8^ c	3.09 d	9143 d	0.31 bc
151-2	3150 c	16.24 b	3275.10 b	6.26 × 10^8^ bc	2.80 d	8760 d	0.29 d
374-1	3699 ab	14.80 c	2955.00 d	7.28 × 10^8^ a	17.69 b	67,136 b	0.32 b
662-2	3095 cd	13.59 d	2879.30 d	6.43 × 10^8^ b	1.45 e	4663 d	0.25 e
903-3	3550 b	14.99 c	3045.90 c	7.08 × 10^8^ a	11.78 c	43,226 c	0.31 bc
LLR059	3651 ab	13.04 e	2117.20 f	5.94 × 10^8^ bc	0.78 f	2744 d	0.36 a
NDCMP49	1996 e	28.56 a	5559.90 a	3.82 × 10^8^ d	68.78 a	139,796 a	0.30 cd
Mean	3235	16.35	3201.90	6.23 × 10^8^	13.50	35,235	0.31
F-test	**	**	*	*	**	**	**
CV%	9.07	2.73	3.34	10.08	4.04	26.41	6.05

* = significantly different at *p* < 0.05, ** = significantly different at *p* < 0.01. Different letters after the mean within a column indicate a significant difference. CV = the coefficient of variation, 1000 GW = 1000 grain weight, GSA = grain surface area, and HI = harvest index.

**Table 4:** Data on chemical composition, soil physics, and climate.

Location	Soil Texture	Total N (%)	Avai. P (mg/kg)	K (mg/kg)	Meters Above Sea Level	Temperature (°C)		Rainfall Amount (mL)
						Maximum	Lowest	
Khon Kaen University Field, Thailand in 2022 (1)	Sandy loam	0.029	5.88	36.95	200	32.21	21.15	1199.40
Khon Kaen Rice Research Center in 2021 (2)	Loamy	0.033	60.00	37.96	187	32.84	21.79	1200.00
Khon Kaen Rice Research Center in 2022 (3)	Loamy	0.033	60.00	37.96	187	32.84	21.79	1000.00
Ban Haet District, Khon Kaen Province, Thailand in 2022 (4)	Sandy	0.019	19.50	25.03	207	32.50	22.30	1243.70

## Data Availability

The data presented in this study are available upon request from the corresponding author.

## Supplementary Materials

The following supporting information can be downloaded at: https://www.mdpi.com/article/10.3390/plants13192713/s1, Figure S1: Rainfall, high-low temperatures at the Khon Kaen Rice Research Center 2021–2022 (A,B), Field Crop Category, Faculty of Agriculture Khon Kaen University (C) and Ban Nong Saeng, Nong Saeng Subdistrict, Ban Haed District, Khon Kaen Province (D); Table S1: Selected rice grain characteristics per spikelet types, grain size, and color of brown rice grain in recombinant inbred lines between Niaw Dam Chaw Mai Pai 49 and LLR059 (Khao Nok); Table S2: Tiller number and panicle number of hybrid varieties between Niaw Dam Chaw Mai Pai 49 and LLR059 (Khao Nok) that were tested in 4 environments, KKRRC 2021, KKRRC 2022, KKU 2022, and BH 2022; Table S3: 1000GW, Grain yield, GSA/1000grains, GSA/ha, Anthocyanin content, and HI of recombinant inbred lines between Niaw Dam Chaw Mai Pai 49 and LLR059 (Khao Nok) that were tested in Khon Kaen Rice Research Center field in 2021; Table S4; 1000GW, Grain yield, GSA/1000grains, GSA/ha, Anthocyanin content, and HI of recombinant inbred lines between Niaw Dam Chaw Mai Pai 49 and LLR059 (Khao Nok) that were tested in Khon Kaen Rice Research Center field in 2022; Table S5: 1000GW, Grain yields, GSA/1000 grains, GSA/ha, Anthocyanin content, and HI of recombinant inbred lines between Niaw Dam Chaw Mai Pai 49 and LLR059 (Khao Nok) that were tested in Agronomy field crop station, Khon Kaen University in 2022; Table S6: 1000GW, Grain yields, GSA/1000 Grains, GSA/ha, Anthocyanin content, and HI of recombinant inbred lines between Niaw Dam Chaw Mai Pai 49 and LLR059 (Khao Nok) that were tested in the farmer field of Ban Nong Saeng, Ban Haet district, Khon Kaen province in 2022.
