# Supplementary material for: Breeding Rice to Increase Anthocyanin Yield Per Area through Small, Black Grain Size and Three Grains per Spikelet
Source: Plants (Basel). 2024 Sep 27;13(19):2713. doi: 10.3390/plants13192713 (PMC11479078; doi:10.3390/plants13192713)
Supplement: Supplementary file 1 [file plants-13-02713-s001.zip › plants-3174735-supplementary.pdf]

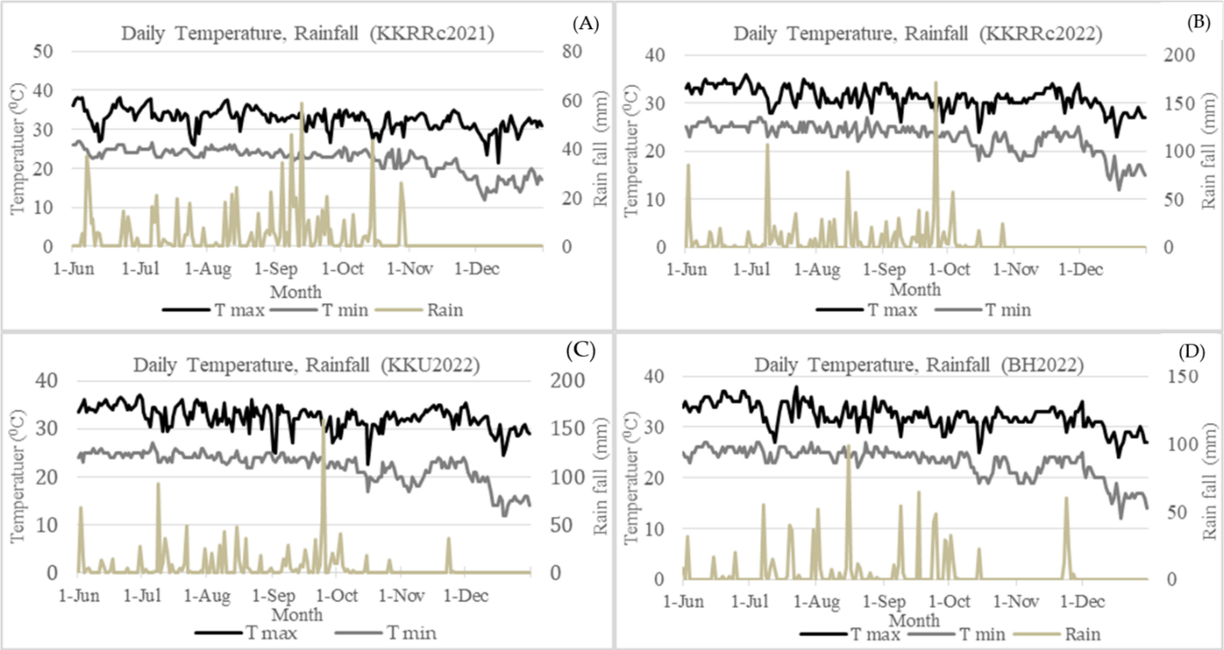


**Supplementary Figure** S1. Rainfall, high-low temperatures at the Khon Kaen Rice Research Center 2021–2022 (A, B), Field Crop Category, Faculty of Agriculture Khon Kaen University (C) and Ban Nong Saeng, Nong Saeng Subdistrict, Ban Haed District, Khon Kaen Province (D).

**Supplementary Table S1.** Selected rice grain characteristics per spikelet types, grain size, and color of brown rice grain in recombinant inbred lines between Niaw Dam Chaw Mai Pai 49 and LLR059 (Khao Nok).

| No. | Lines | Spikelet Types | Grain Size | | | | Color of Brown Rice Grain |
|---|---|---|---|---|---|---|---|
| | | | Grain Length (mm) | Grain Width (mm) | Thickness (mm) | GSA/ grain ($mm^2$) | |
| 1 | 13-1 | 3 | 5.51 | 3.19 | 1.78 | 31.21 | 2 |
| 2 | 145-1 | 3 | 6.38 | 2.53 | 1.52 | 26.62 | 3 |
| 3 | 151-2 | 3 | 6.18 | 3.15 | 1.73 | 32.75 | 3 |
| 4 | 374-1 | 3 | 6.32 | 2.78 | 1.64 | 29.55 | 6 |
| 5 | 662-2 | 3 | 6.40 | 2.64 | 1.64 | 28.79 | 4 |
| 6 | 903-3 | 3 | 6.23 | 2.87 | 1.69 | 30.46 | 6 |
| 7 | LLR059 | 1 | 5.86 | 2.45 | 1.22 | 21.17 | 1 |
| 8 | NDCMP49 | 3 | 9.70 | 3.46 | 2.22 | 55.60 | 7 |

Spikelet types; rice panicles; GSA = grain surface area; color of brown rice grain classified as follows: 1 = white, 2 = light brown, 3 = speckled brown, 4 = brown, 5 = red, 6 = variable purple, 7 = purple.

**Supplementary Table S2.** Tiller number and panicle number of hybrid varieties between Niaw Dam Chaw Mai Pai 49 and LLR059 (Khao Nok) that were tested in 4 environments, KKRRC 2021, KKRRC 2022, KKU 2022, and BH 2022.

| **Lines/ variety** | **Tiller number** | | | | | | | | **Panicle number** | | | | | | | |
|---|---|---|---|---|---|---|---|---|---|---|---|---|---|---|---|---|
| | **KKRRC 2021** | | **KKRRC 2022** | | **KKU 2022** | | **BH 2022** | | **KKRRC 2021** | | **KKRRC 2022** | | **KKU 2022** | | **BH 2022** | |
| 13-1 | 12.30 | b | 12.75 | a | 13.33 | a | 14.25 | bc | 11.17 | b | 11.00 | a | 11.75 | a | 9.96 | a |
| 145-1 | 9.17 | cd | 10.25 | b | 10.08 | cd | 11.92 | d | 8.30 | c-e | 7.00 | bc | 8.25 | c | 9.13 | ab |
| 151-2 | 7.90 | de | 7.50 | c | 10.33 | b-d | 12.29 | d | 7.00 | de | 7.00 | bc | 7.75 | c | 9.58 | a |
| 374-1 | 11.00 | bc | 9.58 | b | 11.75 | a-c | 14.38 | bc | 9.80 | bc | 7.67 | b | 9.08 | bc | 9.50 | ab |
| 662-2 | 9.07 | c-e | 7.58 | c | 9.75 | d | 12.96 | cd | 8.47 | c-e | 6.00 | c | 8.67 | c | 7.96 | bc |
| 903-3-1 | 10.47 | bc | 9.58 | b | 11.83 | a-c | 14.92 | ab | 9.00 | b-d | 7.67 | b | 10.42 | ab | 9.21 | ab |
| LLR059 | 17.40 | a | 12.08 | a | 12.00 | ab | 16.17 | a | 15.43 | a | 10.67 | a | 11.33 | a | 9.96 | a |
| NDCMP49 | 6.73 | e | 4.67 | d | 4.92 | e | 6.92 | e | 6.13 | e | 3.00 | d | 4.17 | d | 6.54 | c |
| Mean | 10.50 | | 9.25 | | 10.50 | | 12.97 | | 9.41 | | 7.50 | | 8.93 | | 8.98 | |
| F-test | ** | | ** | | ** | | ** | | ** | | ** | | ** | | ** | |
| CV% | 12.82 | | 10.62 | | 9.77 | | 6.62 | | 14.65 | | 11.95 | | 9.46 | | 9.84 | |

** = significant difference at $p < 0.01$, * = significant difference at $p < 0.05$, Different letters after the mean within a column showed a significant difference, CV = the coefficient of variation. KKRRC = Khon Kaen Rice Research Center, KKU= Khon Kaen University, BH = Ban Haet district, Khon Kaen province.

**Supplementary Table S3.** 1000GW, Grain yield, GSA/1000grains, GSA/ha, Anthocyanin content, and HI of recombinant inbred lines between Niaw Dam Chaw Mai Pai 49 and LLR059 (Khao Nok) that were tested in Khon Kaen Rice Research Center field in 2021.

| **Lines/ Variety** | **1000GW (g)** | | **Grain yield (kg/ha)** | | **GSA ($cm^2$/1000 grains)** | | **GSA ($cm^2$/ha)** | | **Anthocyanin Content (mg/100g grains)** | | **Anthocyanin content (mg/ha)** | | **HI** | |
|---|---|---|---|---|---|---|---|---|---|---|---|---|---|---|
| 13-1 | 16.45 | b | 4,214 | a | 3,191.40 | b | $8.24 \times 10^8$ | a | 1.96 | ef | 8,267 | e | 0.40 | a |
| 145-1 | 14.70 | c | 2,546 | d | 2,533.00 | d | $4.38 \times 10^8$ | d | 2.62 | df | 6,686 | d | 0.33 | c |
| 151-2 | 16.37 | b | 3,347 | bc | 3,219.40 | b | $6.60 \times 10^8$ | bc | 1.52 | d | 5,105 | d | 0.35 | bc |
| 374-1 | 14.67 | c | 4,012 | a | 2,918.30 | c | $8.00 \times 10^8$ | a | 13.70 | b | 54,795 | b | 0.38 | ab |
| 662-2 | 13.44 | d | 3,104 | cd | 2,629.90 | d | $6.03 \times 10^8$ | c | 1.17 | ef | 3,655 | e | 0.26 | d |
| 903-3 | 15.86 | b | 3,906 | ab | 3,147.40 | b | $7.76 \times 10^8$ | ab | 11.29 | c | 43,843 | c | 0.39 | ab |
| LLR059 | 12.95 | d | 4,341 | a | 2,105.50 | e | $7.04 \times 10^8$ | a-c | 0.34 | f | 1,493 | e | 0.38 | ab |
| ULR416 | 28.75 | a | 1,663 | e | 5,510.50 | a | $3.19 \times 10^8$ | d | 53.79 | a | 89,521 | a | 0.35 | bc |
| Mean | 16.65 | | 3,391 | | 3,156.90 | | $6.40 \times 10^8$ | | 10.79 | | 26,671 | | 0.36 | |
| F-test | ** | | ** | | ** | | ** | | ** | | ** | | ** | |
| CV% | 3.50 | | 10.33 | | 2.56 | | 11.48 | | 4.31 | | 13.67 | | 5.64 | |

** = significant different at $p < 0.01$. Different letters after the mean within a column showed a significant difference. CV = the coefficient of variation, 1000 GW = 1000 grain weight, GSA = Grain surface area, and HI = harvest index.

**Supplementary Table S4.** 1000GW, Grain yield, GSA/1000grains, GSA/ha, Anthocyanin content, and HI of recombinant inbred lines between Niaw Dam Chaw Mai Pai 49 and LLR059 (Khao Nok) that were tested in Khon Kaen Rice Research Center field in 2022.

| **Lines/ Variety** | **1000GW (g)** | | **Grain yield (kg/ha)** | | **GSA ($cm^2$/1000 grains)** | | **GSA ($cm^2$/ha)** | | **Anthocyanin content (mg/100g grains)** | | **Anthocyanin content (mg /ha)** | | **HI** | |
|---|---|---|---|---|---|---|---|---|---|---|---|---|---|---|
| 13-1 | 16.82 | b | 3,348 | a | 3,094.30 | bc | $6.16 \times 10^8$ | a | 1.29 | ef | 4,335 | d | 0.35 | a |
| 145-1 | 11.72 | e | 2,815 | bc | 2,575.80 | d | $6.21 \times 10^8$ | a | 2.25 | de | 6,335 | d | 0.27 | bc |
| 151-2 | 16.11 | b | 2,634 | c | 3,304.30 | b | $5.45 \times 10^8$ | a | 3.14 | d | 8.279 | d | 0.26 | bc |
| 374-1 | 14.45 | c | 3,271 | ab | 2,918.90 | c | $6.61 \times 10^8$ | a | 14.40 | b | 47,104 | b | 0.28 | b |
| 662-2 | 13.59 | d | 2,720 | c | 3,008.90 | c | $6.01 \times 10^8$ | a | 1.41 | ef | 3,848 | d | 0.24 | c |
| 903-3 | 15.12 | c | 3,020 | a-c | 2,980.50 | c | $5.96 \times 10^8$ | a | 8.75 | c | 26,314 | c | 0.28 | b |
| LLR059 | 13.04 | d | 3,390 | a | 2,108.30 | e | $5.48 \times 10^8$ | a | 0.85 | f | 2,897 | d | 0.35 | a |
| ULR416 | 25.06 | a | 1,522 | d | 5,606.70 | a | $3.40 \times 10^8$ | d | 65.49 | a | 99,829 | a | 0.26 | bc |
| Mean | 15.74 | | 2,840 | | 3,199.70 | | $5.66 \times 10^8$ | | 12.20 | | 24,868 | | 0.29 | |
| F-test | ** | | ** | | ** | | ** | | ** | | ** | | ** | |
| CV% | 2.66 | | 9.50 | | 4.22 | | 12.40 | | 5.42 | | 17.83 | | 6.50 | |

** = significant different at $p < 0.01$. Different letters after the mean within a column showed a significant difference. CV = the coefficient of variation, 1000 GW = 1000 grain weight, GSA = Grain surface area, and HI = harvest index.

**Supplementary Table S5.** 1000GW, Grain yields, GSA/1000 grains, GSA/ha, Anthocyanin content, and HI of recombinant inbred lines between Niaw Dam Chaw Mai Pai 49 and LLR059 (Khao Nok) that were tested in Agronomy field crop station, Khon Kaen University in 2022.

| **Lines/ Variety** | **1000GW (g)** | | **Grain yield (kg/ha)** | | **SSA ($cm^2$/1000 grains)** | | **GSA ($cm^2$/ha)** | | **Anthocyanin content (mg/100g grains)** | | **Anthocyanin content (mg /ha)** | | **HI** | |
|---|---|---|---|---|---|---|---|---|---|---|---|---|---|---|
| 13-1 | 16.60 | b | 4,536 | a | 3088.10 | c | $8.46 \times 10^8$ | a | 1.71 | ef | 7,770 | c | 0.39 | a |
| 145-1 | 13.95 | d | 3,327 | c | 2747.20 | e | $6.53 \times 10^8$ | b | 4.21 | d | 13,936 | d | 0.36 | ab |
| 151-2 | 17.00 | b | 3,208 | c | 3315.60 | b | $6.25 \times 10^8$ | b | 3.29 | d | 12,624 | d | 0.27 | de |
| 374-1 | 15.41 | c | 4,186 | ab | 3065.60 | cd | $8.34 \times 10^8$ | a | 28.98 | b | 121,253 | b | 0.32 | bc |
| 662-2 | 13.61 | d | 3,883 | b | 2887.00 | de | $8.21 \times 10^8$ | a | 2.11 | e | 8,189 | d | 0.24 | e |
| 903-3 | 14.96 | c | 4,167 | ab | 3036.40 | cb | $8.48 \times 10^8$ | a | 17.40 | c | 72,582 | c | 0.30 | cd |
| LLR059 | 13.31 | d | 3,817 | b | 2106.60 | f | $6.03 \times 10^8$ | b | 1.03 | f | 3,899 | d | 0.39 | a |
| ULR416 | 30.90 | a | 2,337 | d | 5610.70 | a | $4.24 \times 10^8$ | c | 108.17 | a | 252,567 | a | 0.30 | cd |
| mean | 16.97 | | 3,683 | | 3,232.10 | | $7.07 \times 10^8$ | | 31.54 | | $6.90 \times 10^6$ | | | |
| F-test | ** | | ** | | ** | | ** | | ** | | ** | | | |
| CV% | 2.70 | | 6.20 | | 3.39 | | 6.30 | | 3.36 | | 6.28 | | | |

** = significant different at $p < 0.01$. Different letters after the mean within a column showed a significant difference. CV = the coefficient of variation, 1000 GW = 1000 grain weight, GSA = Grain surface area, and HI = harvest index.

**Supplementary Table S6.** 1000GW, Grain yields, GSA/1000 Grains, GSA/ha, Anthocyanin content, and HI of recombinant inbred lines between Niaw Dam Chaw Mai Pai 49 and LLR059 (Khao Nok) that were tested in the farmer field of Ban Nong Saeng, Ban Haet district, Khon Kaen province in 2022.

| Lines/ Variety | 1000GW (g) | | Grain yield (kg/ha) | | GSA ($cm^2$/1000 grains) | | GSA ($cm^2$/ha) | | Anthocyanin content (mg/100g grains) | | Anthocyanin content (mg/ ha) | | HI | |
|---|---|---|---|---|---|---|---|---|---|---|---|---|---|---|
| 13-1 | 16.94 | b | 3,270 | a | 3,111.80 | bc | $6.00 \times 10^8$ | ab | 1.58 | e | 5,267 | de | 0.31 | ab |
| 145-1 | 13.25 | e | 2,931 | a-c | 2,790.20 | e | $6.18 \times 10^8$ | ab | 3.26 | d | 9,614 | d | 0.29 | bc |
| 151-2 | 16.49 | b | 3,410 | a | 3,261.10 | b | $6.74 \times 10^8$ | a | 2.62 | d | 9,033 | d | 0.29 | bc |
| 374-1 | 15.67 | c | 3,325 | a | 2,917.10 | de | $6.19 \times 10^8$ | ab | 13.68 | b | 45,393 | b | 0.30 | ab |
| 662-2 | 14.71 | d | 2,672 | bc | 2,991.40 | cd | $5.48 \times 10^8$ | b | 1.10 | e | 2,958 | e | 0.25 | cd |
| 903-3 | 15.35 | c | 3,108 | ab | 3,019.50 | cd | $6.11 \times 10^8$ | ab | 9.73 | c | 30,166 | c | 0.27 | d |
| LLR059 | 12.53 | f | 3,056 | ab | 2,148.60 | f | $5.23 \times 10^8$ | bc | 0.89 | e | 2,687 | e | 0.32 | a |
| ULR416 | 30.61 | a | 2,461 | c | 5,511.90 | a | $4.43 \times 10^8$ | c | 47.67 | a | 117,268 | a | 0.29 | bc |
| Mean | 16.94 | | 3,029 | | 3,218.90 | | $5.80 \times 10^8$ | | 10.07 | | 27,798 | | 0.29 | |
| F-test | ** | | * | | ** | | ** | | ** | | ** | | ** | |
| CV% | 2.03 | | 10.27 | | 2.94 | | 10.18 | | 4.58 | | 9.33 | | 5.40 | |

** = significant different at $p < 0.01$. Different letters after the mean within a column showed a significant difference. CV = the coefficient of variation, 1000 GW = 1000 grain weight, GSA = Grain surface area, and HI = harvest index.
